# Enhancing Stem Cell Therapy for Cartilage Repair in Osteoarthritis—A Hydrogel Focused Approach

**DOI:** 10.3390/gels7040263

**Published:** 2021-12-14

**Authors:** Yisi Liu, Meng Wang, Yixuan Luo, Qianyi Liang, Yin Yu, Fei Chen, Jun Yao

**Affiliations:** 1 Collaborative Innovation Center of Regenerative Medicine and Medical Biological Resources Development and Application, Guangxi Medical University, Nanning 530021, China; ys.liu@siat.ac.cn; 2 Center for Materials Synthetic Biology, CAS Key Laboratory of Quantitative Engineering Biology, Shenzhen Institute of Synthetic Biology, Shenzhen Institute of Advanced Technology, Chinese Academy of Sciences, Shenzhen 518055, China; meng.wang1@siat.ac.cn (M.W.); yx.luo@siat.ac.cn (Y.L.); qy.liang@siat.ac.cn (Q.L.); 3 Guangxi Key Laboratory of Regenerative Medicine, Guangxi Medical University, Nanning 530021, China; 4 International Joint Laboratory on Regeneration of Bone and Soft Tissues, Guangxi Medical University, Nanning 530021, China; 5 Bone and Joint Surgery Unit, The First Affiliated Hospital of Guangxi Medical University, Nanning 530021, China

**Keywords:** stem cell, hydrogel, osteoarthritis, cartilage repair

## Abstract

Stem cells hold tremendous promise for the treatment of cartilage repair in osteoarthritis. In addition to their multipotency, stem cells possess immunomodulatory effects that can alleviate inflammation and enhance cartilage repair. However, the widely clinical application of stem cell therapy to cartilage repair and osteoarthritis has proven difficult due to challenges in large-scale production, viability maintenance in pathological tissue site and limited therapeutic biological activity. This review aims to provide a perspective from hydrogel-focused approach to address few key challenges in stem cell-based therapy for cartilage repair and highlight recent progress in advanced hydrogels, particularly microgels and dynamic hydrogels systems for improving stem cell survival, retention and regulation of stem cell fate. Finally, progress in hydrogel-assisted gene delivery and genome editing approaches for the development of next generation of stem cell therapy for cartilage repair in osteoarthritis are highlighted.

## 1. Introduction

Osteoarthritis (OA) is a chronic degenerative joint disease characterized by pain and stiffness [1]. It is a progressive disease of the synovial joints, resulting in the destruction of bone, articular cartilage and subchondral bone [2,3]. Since articular cartilage has limited innate self-healing ability, the treatment for cartilage injury caused by osteoarthritis has been a major clinical challenge. Recent studies have shown that there is a close link between inflammation and progression of osteoarthritis, and the main involved factors include reactive oxygen species (ROS), pro-inflammatory cytokines, matrix degrading enzymes, nitric oxide and biomechanical stress. Therefore, identifying early inflammatory events and providing timely treatment for inflammation will attenuate the major symptoms of OA patients [4].

In the early stages of OA development, conservative nonsurgical management of OA and pharmacological therapies can be effective in relieving pain, but are not able to reverse cartilage degeneration [5]. For example, steroid and non-steroidal anti-inflammatory drugs (e.g., corticosteroids) as well as injection of hyaluronic acid (HA) can alleviate the OA symptoms, but had no suppression on the progression o of OA. For patients with severe joint injuries or osteoarthritis that do not respond to conservative treatment, surgical treatments such as joint replacement and osteotomy are recommended but long-term outcomes for patients can vary significantly [5,6].

Hydrogels are crosslinked hydrophilic polymer networks that exhibit the properties of elastic solids with deformability and softness. Owing to their high-water content, biocompatibility and permeability to a wide range of biological molecules, hydrogels are being intensively used in tissue engineering, drug delivery and biological research. Hydrogel scaffolds with high strength and resilience can be achieved by chemical modification or through three-dimensional weaving technology, suitable for use in repairing or regenerating tissues and quickly restoring the biomechanical function of tissues [7,8,9,10].

Stem cells have been extensively studied in preclinical stages due to their anti-inflammatory, immunoregulatory and regenerative properties [11], and are expected to treat OA [12,13]. For example, intra-articular injection of adipose mesenchymal stem cells (MSCs) not only alleviated the degeneration of articular cartilage, but also decreased the International Association for Osteoarthritis Research (OARSI) and Mankin scores, and reduced MMP13 expression at different stages in the rabbit model of OA [14]. In an experimental trial, researchers compared the clinical outcomes of the transplantation of first-generation autologous chondrocytes and bone marrow mesenchymal stem cells (BM-MSCs) for cartilage repair in 36 patients in each group, they found life quality of all patients was improved and there was no significant difference between these two cell-types groups [15]. However, there are various sources of MSCs used for tissue engineering. The MSCs from the different sources have their own advantages and disadvantages. For example, bone marrow mesenchymal stem cells (BMSCs) have better chondrogenic capability compared to adipose tissue-derived MSCs [16]. Thus, MSC-based therapy is promising to provide an effective and less invasive method to repair articular cartilage defects. Despite great potential of stem cell therapies for cartilage defect and OA treatment, many challenges still exist. For instance, MSCs used in the treatment of bone and articular cartilage disease through systemic administration means that the application of this therapy implies and requires efficient migration and homing to the target site; however, experiments have proved that this process is relatively inefficient because only a small part of MSCs administered systematically can actually reach the target tissue; the percentage of MSCs in the different tissues was estimated between 0.1% and 2.7% [17]. While local administration provides direct access to the disease site and generally results in better therapeutic outcomes, insufficient retention and survival of transplanted MSCs at the site of administration still hinder their therapeutic efficacy [18]. Besides, cell targeting [19], injection device properties (needle size/geometry) [18] and pathological microenvironment [20] on cells are other challenges associated with local administration. In this review we highlight few approaches of using advanced hydrogels to enhance the efficacy of MSCs for treatment of cartilage defects and osteoarthritis.

## 2. Hydrogel Encapsulation Can Prolong the Retention and Survival Time of MSCs In Vivo

### 2.1. Prolong MSC Survival

To increase the survival of MSCs at the infarction site by local administration [21], similar approaches as the delivery of conventional drugs by encapsulation for OA treatment and cartilaginous diseases can be adopted, since stem cells could be regarded as “living drugs”. Inspired by this, microcapsules are used for encapsulating MSCs to improve the cell survival and therapeutic efficiency. For example, leveraging microfluidic platforms, living cells can be encapsulated inside uniform alginate hydrogel microparticles, the number of living cells in the microcapsules can be controlled and 65% of the cells encapsulated in the alginate microparticles were still viable after one week [22]. In another study, a biocompatible, self-hardening silanized-hydroxypropylmethylcellulose (Si-HPMC) hydrogel was made by using a droplet microfluidics technique, and the survival rate of human adipose derived stromal/stem cells (hASCs) in Si-HPMC particles remained above 70% after 2 weeks in vitro [23]. It is worth noting that these biomaterials used for encapsulating cells still have efficient diffusion of nutrients, such as vitamins and glucose, which are critical for maintaining the survival of encapsulated cells. Therefore, injectable biomaterials microbeads can not only prevent cells from the shear force generated in delivery process, but form a strong niche for the centralized distribution of paracrine factors in the injection site [24,25]. Moreover, MSC-laden hydrogel microbeads can inhibit inflammation and induce in vitro regeneration of patient-derived cartilage, which can further promote the healing of endogenous cartilage regeneration [26]. However, for functional cartilage regeneration, the microgel needs to be able to effectively bind to the surrounding tissue to achieve better integration between neotissue and native tissue [27]. Recently, researchers designed and developed a procedure for attaching the microgel to the surrounding tissue. Experiments demonstrated rapid fixation of a cartilage defect by injecting the cell-laden microgel. This strategy is favorable for clinical translation compared to bulk hydrogel because it not only preserves encapsulated cell viability, but also enhances human bone marrow mesenchymal stem cells (hBMSC) chondrogenesis [28]. In another recent study, multifunctional hyper-branched poly (ethylene glycol) diacrylate (PEGDA) could rapidly cross-link with thiolated hyaluronic acid (HA) under physiological conditions to achieve gelation, facilitates in situ cell encapsulation and supports cell survival, self-renewal and differentiation [29,30]. The high proportion of acrylate groups in the polymer chain provides a wide range of tunability for the hydrogel system, which can be optimized to regulate the behavior of stem cells [31]. On the other hand, the incorporation of nanoparticles/nanofibers into hydrogels provide another way to regulate cell behavior and hydrogel properties, for example, anti-inflammatory drugs can be loaded into nanoparticles and sustained release can be achieved in bulk hydrogel. In addition, short electrospun nanofiber can be introduced to improve the mechanical properties of hydrogel.

### 2.2. Increase Retention

**Figure 1:**
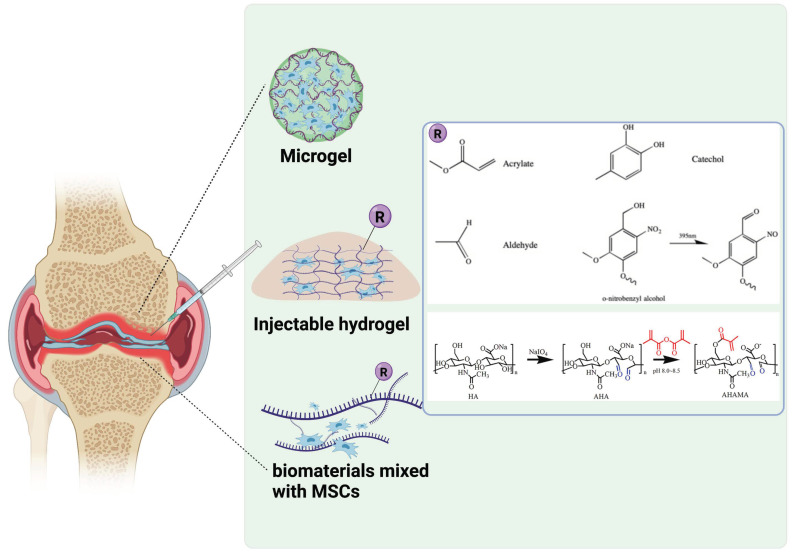
Schematic description of utilizing injectable microgels and bioadhesive hydrogels to increase cell survival and retention. (Created with BioRender.com).

While biomaterials-based microgel could effectively encapsulate MSCs and improve long-term cell survival, insufficient MSCs retention in the injected defect site still represent a main barrier against successful outcomes of MSCs therapy for cartilage defect. Until now, a variety of means have been put forth to overcome this issue. Figure 1 shows different approaches utilized to prolong cell survival and retention at the tissue defect site. For example, thermosensitive hydrogels have been widely used to improve cellular retention with simple operation. A recent study has constructed an injectable chitosan/glycerol phosphate sodium/cellulose nanocrystal (CS/GP/CNC) for encapsulating human umbilical cord-mesenchymal stem cells (hUCMSCs). The introduction of 2% CNC increased the compressive modulus of gels from ~0.1 MPa to 2.3 MPa. Besides, because excess GP in the gel system that does not participate in the gel process dissolves rapidly in PBS, the composite gels exhibited a fast degradation rate, with a 50% weight loss in 10 days [32]. However, the gelation speed of this system is relatively low.

Another family of widely used bioadhesive is based on aldehyde functionalized polymers [33,34]. Recently Chen et al. fabricated an injectable and adhesive hyaluronic acid (AHAMA) hydrogel modified by aldehyde groups and methacrylate on the polysaccharide backbone with multiple anchoring mechanisms. According to the results of modified O ‘Driscoll histological score evaluation of cartilage repair at 4 and 12 weeks and mean density of collagen type II staining, the therapeutic group had nearly two times the repair effect compared with the non-therapeutic group. The expression of AVAN, COL 1 and other genes was nearly 5–10 times higher than the control group, combined with the nearly two fold increase of various indicators for cartilage repair in immunohistochemical results. Thus the authors suggested that AHAMA can promote the proliferation and migration of BMSCs and significantly improve cartilage defects [34]. However, high degree of substitution of aldehyde groups along the polymer backbone could be toxic to encapsulated cells. Similar results have been found in polydopamine modified chondroitin sulfate-polyacrylamide (PDA-CS-PAM) hydrogel in a growth-factor free system for cartilage regeneration [35].

Hyaluronic acid (HA) hydrogels have a promising perspective for regeneration of cartilage since they are the component of the cartilage. It was reported that photopolymerized HA hydrogels can induce the chondrogenesis of MSCs [36]. Photo-induced free radical polymerization is another fast and efficient strategy to form interconnected hydrogel network, based on a photoinduced imine crosslinking (PIC) hydrogel capable of generating aldehyde group under light irradiation, which subsequently adheres to the surround tissue. Zhu and colleagues mixed PIC pre-gel with autologous platelet-rich plasma (PRP) and formed an injectable hydrogel glue. This hydrogel not only achieved controllable release of growth factors, but also improved the cartilage adhesion and integration of PRP-loaded hydrogel in a light-controlled manner [37]. Similar results were also observed in the restoration of skin tissue [38]. In another study, stem cell-derived exosomes (SC-Exos) was encapsulated in PIC hydrogel to form an articular cartilage tissue patch, which showed good biocompatibility, tissue adhesion, as well the exact matching of tissue patch and irregular tissue defect [39].

## 3. Guiding MSCs Fate via Viscoelastic Biomaterials

The extracellular matrix (ECM) provides structural, biochemical, and other support to surrounding cells, maintains tissue hemostasis and promotes constructive remodeling of tissue. Although the mechanism by which ECM promotes constructive tissue remodeling remains unclear, it has been gradually recognized that time-dependent mechanics performances, namely viscoelastic properties (e.g., stress relaxation and creep) of ECM are a key player in the cell fate decision healing process [40]. Thus, to mimic or even recapitulate cartilage and/or bone tissues in vitro, the recapitulation of its ECM viscoelastic microenvironment is of great importance [41]. Hydrogel, a crosslinked hydrophilic polymer that possess similar structural network and water content with ECM, can be designed with specific viscoelasticity that toward target tissues for controlling the cellular behaviors [42]. Different crosslinking mechanisms (e.g., physical interactions, supramolecular interaction and dynamic covalent interactions) for synthesizing viscoelastic hydrogels and the underlying mechanobiology mechanisms have been reviewed elsewhere [43]. Here we will highlight the influence of dynamically mechanical microenvironment created by various viscoelastic hydrogels, including supramolecular networks, interpenetrating network (IPN) and dynamic bonds formed networks, on the fate of MSCs in the osteochondral restore and regeneration.

**Figure 2:**
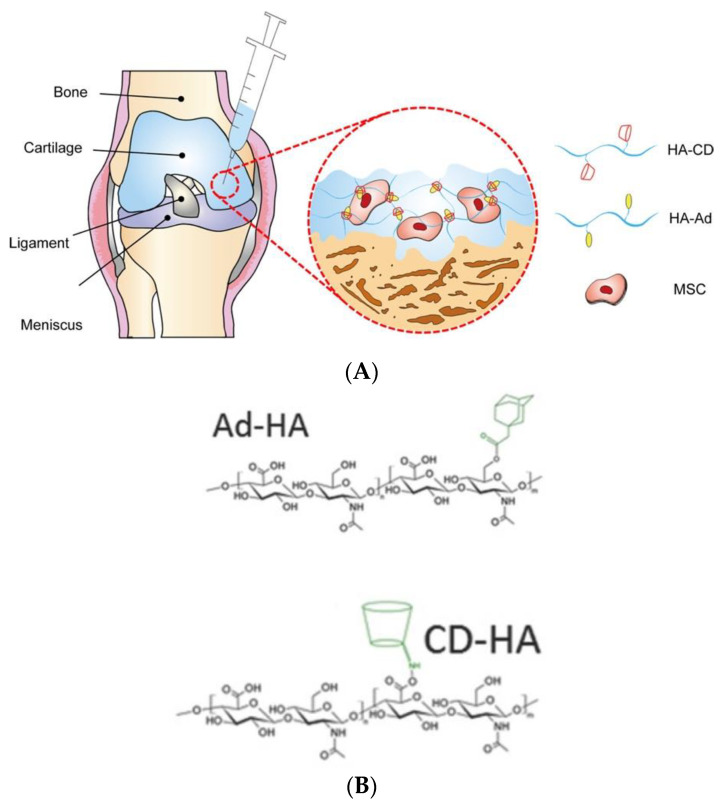

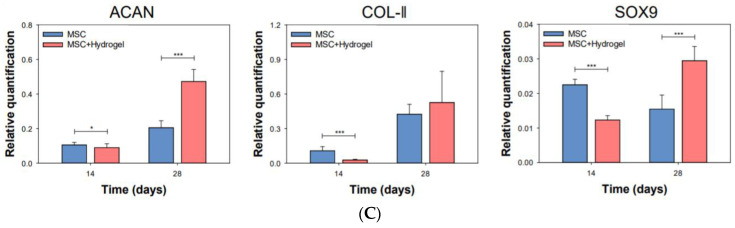
(**A**) MSCs-loading supramolecular hydrogels applied for cartilage tissue regeneration; (**B**) Chemical structures of injectable HA hydrogels crosslinked by host-guest interaction between β -Cd and Ad, (**C**) Supramolecular hydrogels carrying MSCs showed better chondrogenic efficiency than MSCs only group, according to the relative quantification of mRNA levels for chondrogenic markers at days 14 and 28 (*n* = 4, * *p* < 0.05, *** *p* < 0.001) [44].

Supramolecular networks are formed by reversible and non-covalent intermolecular interactions and allow to tailor the mechanic properties of hydrogel that recapitulate the dynamic and viscoelastic behaviors of ECM [44]. One of the most used mechanisms for synthesizing supramolecular biomaterials is through interactions between molecules of host and guest, which has been adopted to improve the regeneration of both hyaline cartilage and subchondral bone. For instance, hyaluronate (HA)-based supramolecular hydrogels, prepared by the reaction between the host molecule β-cyclodextrin (CD) and guest molecule of adamantane-modified HA (ad), exhibited higher efficiency of chondrogenic differentiation of MSCs and the deposition of ECM than the MSCs only group (Figure 2A–C) [44]. However, this type of molecule interaction has weak bonds, and its mechanical properties are lower than the native cartilage. Feng et al. developed a viscoelastic hydrogel with interpenetrating networks (IPN) for cartilage regeneration, in this hydrogel, supramolecular host-guest macromer (HGM) networks between the gelatinous aromatic residues and β-cyclodextrin (β-CD) hold fast sol-gel transition ability and give hydrogel cartilage-like resilient property [45], at the same time acrylated β-cyclodextrin (β-CD) monomers is photo-crosslinkable, which may provide a strong covalent network and hence increase the mechanical performances of the produced viscoelastic hydrogel. The generated dynamical hydrogel showed better chondrogenesis of MSCs than conventional chemically crosslinked gelatin hydrogels under both in vivo and in vitro condition [46]. Indeed, this viscoelastic hydrogel with IPN structure is another strategy to enhance the specific cell lineage differentiation of BMSCs [47].

The IPN viscoelastic hydrogel with controlled stress relaxation property is due to the introduction of energy dissipative components (provide weak network) into mechanically strong system. In this type of hydrogels, weak network can unbind and allow subsequent covalent network flow under stress or strain, and they can rebind or reform following flow to dissipate energy, contributing to a dynamic mechanical niche that cells reside. For instance, Li et al. developed a composite hydrogel with improved mechanical strength and unique stress relaxation behavior by combining physical cross-linked gellan gum (GG) with chemical cross-linked polyethylene glycol diacrylate (PEGDA). Cell culture results showed that after one week of co-culture, GG could not remain stable in the medium due to brittleness and insufficient mechanical properties, while the BMSC clusters cultured on GG/PEGDA IPN hydrogel tended to have larger areas and more irregular morphologies than those on PEGDA hydrogel, as can be seen from the cell apoptosis data, the value of GG/PEGDA IPN is less than that of GG and PEDGA alone, and the PEDGA apoptosis ratio has reached to 20% on the third day. Therefore, GG/PEGDA IPN possess complex mechanical properties, better resemble the microenvironment of native ECM, and proved that DN hydrogel enhances the chondrogenic differentiation of BMSCs in vivo. [47] Similarly, Chaudhuri and colleagues found that arginine-glycine-aspartic acid (RGD) modified alginate/PEG hydrogels with faster relaxation lead increased cell spreading and proliferation of fibroblast, as well as the enhanced osteogenesis of MSCs [48]. Although the target application in above study was not for cartilage regeneration, this work provides a simple strategy to tuning stress relaxation of hydrogels for regulating MSCs behavior and thus holds promise for osteochondral regeneration. It is worth noting that supramolecular hydrogel is also able to release drugs in a long-term and sustained way, thus if cytokines capable of inducing MSCs to undergo chondrogenic are encapsulated in these supramolecular hydrogels, the cartilage repair efficiency could be further promoted. Moreover, compared to chemical crosslinked hydrogels, supramolecular viscoelastic hydrogels facilitate cell infiltration and migration [49], which is beneficial to recruit the endogenous cells to participate in tissue remodeling process. However, design and development of a hydrogel with appropriate stress relaxation kinetics which can adapt the tissue remodeling processes in vivo is still a big challenge.

Dynamic covalent chemistry is another promising way to modulate cell fate via its tunable and adaptable viscoelastic characteristics [50]. Among different dynamic chemical bonds utilized to form viscoelastic hydrogels, Imine (Schiff bases, hydrazones and oximes) represent a widely investigated family, which formed by the reaction of a nucleophilic amines and an electrophilic carbonyl group. For instance, Anseth group developed a hydrazine crosslinked poly (ethylene glycol) hydrogels with tunable viscoelastic properties and studied the covalent adaptable network in cartilage tissue engineering. They found that highly adaptive linking improved cellular structures (proliferation, ECM deposition, et al.), but slowly relaxed cross-linking was also required to achieve high quality new cartilage tissue [51,52]. Besides the temporally evolving properties capable of directing cell behavior provided from dynamic materials, dynamic covalent hydrogels also enhance stem cell therapy for cartilage regeneration including as minimally invasive injectable cell delivery vehicles, bioinks for 3D printing [43]. To advance the field of dynamic covalent hydrogels and their application for cartilage tissue engineering, more research should focus on standardizing methods to characterize the viscoelastic properties, especially in presence of stem cells and in vivo condition.

## 4. Engineering MSCs Behavior through Materials-Mediated Gene Delivery and Gene Editing

As was stated in above section, encapsulating MSCs into tissue adhesive hydrogels or microgels has been demonstrated to be an effective strategy to prolong MSC survival and facilitate the adhesion of neotissue to host tissue; however, the proliferation and differentiation of MSCs may be uncontrolled in situ after implantation without defined exogeneous factors. In this regard, compared with top-down tissue engineering approaches, in which cell fate is guided by scaffolds and signals, a bottom-up approach that directly regulates stem cell behavior through gene delivery holds promise to develop more effective cell-carrying structures for cartilage repair. Historically, incorporation of growth factors to the scaffold is one of the most widely used methods to induce MSCs differentiation. However, due to the short half-life of growth factors, treatment is usually administered in vivo at extra-physiological concentrations, which can lead to potential off-target effects and cytotoxicity. To circumvent this issue, gene delivery has been proposed as an alternative approach to modulate cellular behavior.

**Figure 3:**
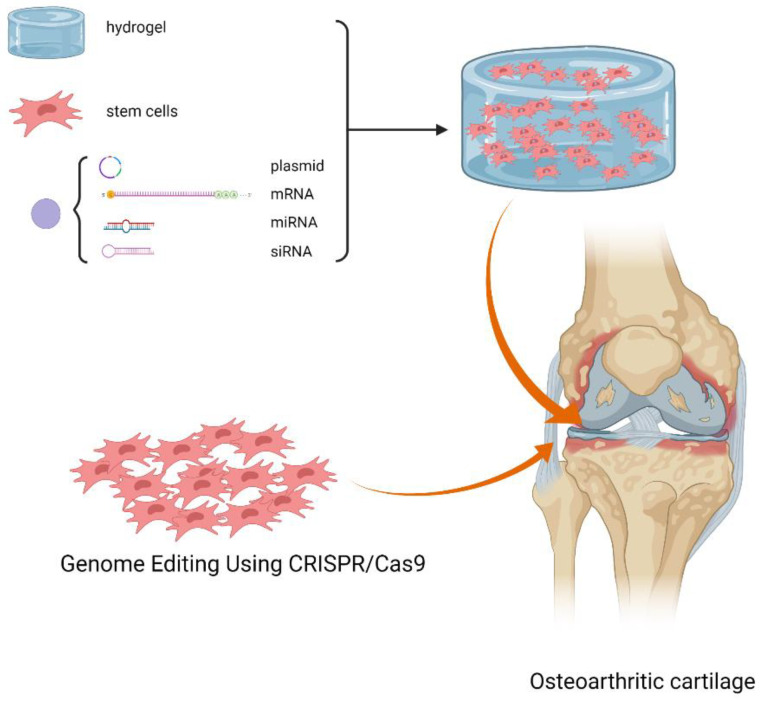
Schematic description of materials-mediated gene delivery and genetically engineered MSCs for cartilage repair and osteoarthritis. (Created with BioRender.com).

Genes (pDNA, mRNA, lncRNA and siRNA) can be administered into host tissue via direct in vivo injection or indirect in vitro transduction. For in vitro transduction, foreign genes can be introduced into targets cells either by virus or non-viral vector such as cationic liposomes and polymeric or inorganic materials. For detailed information about selection of appropriate delivery vehicles, readers are referred to recent review articles [53]. Here we focus our discussion on direct delivery using the combination of vectors and matrix. Figure 3 shows a schematic description of materials-mediated gene delivery and genetically engineered MSCs for cartilage repair and osteoarthritis.

In the delivery system, the vector acts as the bridge of the exogenous target gene entering into the cell to induce the in situ regeneration of cartilage, which directly determines the success or failure of the treatment. However, direct injection of the carrier into the joint cavity is imprecise and transient because the carrier is not easily accessible to the target site due to dilution of the joint fluid [54]. Thus, transgenic expression may occur at other sites leading to harmful side effects, such as immune response and synovial cartilage formation [55] Recent studies have shown that stent-binding gene therapy strategies were able to achieve direct, local, and continuous nucleic acid delivery from the scaffold to ensure efficient and durable cell transfection, and the choice of vectors has a direct impact on the activity of gene products [56,57]. To solve these problems, researchers developed gene-activated matrices (GAMs), which incorporated the gene complex directly into the scaffold by introduction of the vector during scaffold preparation or incorporation of the vector into the assembled scaffold so that it could not be rapidly degraded and engulfed in synovial fluid and offered a spatial confinement of the delivered genes that cells are transfected in situ [58]. The use of GAMs is able to regulate cellular fate and/or affect pathological environments due to its microenvironment-adaptable chemical features, which is conducive to the formation of neotissue in the cartilage defects and the regulation of micro-milieu that stem cells resides.

As GAMs for cartilage repair, non-viral gene delivery is a promising option to recombinant proteins and viral gene transduction in orthopedic tissue engineering. In exploring the use of gene therapy as an alternative strategy for recombinant protein delivery, plasmid DNA (pDNA) based GAMs that encodes growth factors is of interest. The pDNA is first loaded in a polymer matrix and subsequently released to transfect surrounding cells for further sustained and local expression of desired factors [59]. However, it still has some limitations. Although non-viral delivery provides sufficient safety, it is possible to insert to the natural genome and causing gene mutations, which hinder pDNA GAMs successful application in clinical practice. Currently, researchers have turned eyes to the RNA GAMs including siRNA, miRNA, and mRNA-GAMs, which have superior gene transfer capabilities [59] and fine transcription in the cytoplasm [60]. For example, doxycycline-inducible IL-1 receptor antagonist (IL-1Ra) transgene activated cartilage-derived matrix (CDM) can induce the chondrogenic differentiation and further endochondral ossification, which are independent of and synergistic with exogenous growth factors [61]. For the formation of more complex osteochondral tissue, spatial organization of different GAMs and the simultaneous differentiation of chondrogenic and osteogenic via site-specific transduction of a single MSCs population is of importance [62].

In addition, a growing number of studies have shown that the incidence of OA is related to genetic problems [63], but it is difficult to isolate these pathogenic factors from the environmental influences [64]. Therefore, the occurrence of osteoarthritis is considered to be associated with the inflammatory signaling pathways, which can be fine-tuned by using gene-activated materials at the gene level [62]. The clustered regularly interspaced short palindromic repeat (CRISPR)-CRISPR-associated (Cas) endonuclease system is one of the emerging nuclease platforms [65]. In the past decade, researchers have made great strides in genome-editing technologies consisting of CRISPR/Cas9. Using a single non-sequence-specific protein bound to a small guide RNA molecule, specific genes in various living systems, even mammalian organism, can be efficiently and accurately regulated [66,67]. Some studies have reported that CRISPR/Cas9-mediated gene editing can alter multiple inflammatory signal transduction in OA joint tissues. For example, CRISPR/Cas9-adeno-associated virus (AAV) complex have successfully reduced the expression of several target genes in the knee joints, including nerve growth factor (NGF), interleukin-1β (IL-1β) and MMP13, with benefits including both pain relief and structural improvements. Furthermore, specific and histological analyses revealed that multiplex gene editing did not accelerate the progress of post-traumatic OA (PTOA) as rapidly as ablation of NGF alone did. The quantification of AC degradation by OARSI scoring confirmed that the multiplexing group had less destructive changes [68].

Additionally, the identification of promising drug targets could be discovered based on the genetic and epigenetic alternations in OA, potential biomarkers for the diagnosis, prognosis, drug response and further feasible therapeutic strategies for OA treatment could be approached [68]. Furthermore, the development of genome editing techniques has led to the development of ‘designer cell’, including modification of receptors, gene networks or transgenes, laying the foundation for new cell therapies [20]. For example, it has shown that CRISPR-Cas9 engineered stem cells have a synthetic genetic circuit which can respond in an auto-regulated, feedback-controlled manner, and express biological drugs against interleukin-1 (IL-1) or tumor necrosis factor A (TNF-A) [69]. Inflammatory cytokines are the most important compounds involved in the pathogenesis of OA [70], and they imbalance the homeostasis of the joint tissue by promoting catabolic and destructive processes [71]. Therefore, blocking the signaling pathways associated with inflammatory factors is a promising path to ameliorating the pathological environment. A virus expressing the CRISPR/Cas9 component is a strategy that can be used for adjusting the inflammatory milieu of OA joint. For example, it was injected into the OA joint to target some of the genes encoding associated inflammatory factors, and successfully blocked inflammatory signaling pathways [68]. We briefly summarized the different MSCs behavior with a different delivery method in Table 1.

However, the integration of CRISPR-mediated modulation of inflammatory microenvironment and the biomaterials-based scaffold intervened differentiation of stem cells have rarely been reported. If we can combine these two parts together, it would be possible to regulate cellular fate and/or affect pathological environments simultaneously, and thus enhance cartilage repair. In short, gene activated materials have great prospects for next generation of stem cell-based therapies for treatment of OA by regulating the inflammatory signaling pathways and the fate of stem cells.

## 5. Conclusions and Future Prospects

Mesenchymal stem cells (MSCs) hold great potential in developing tissue engineered constructs for cartilage regeneration. A critical factor in determining the success of such constructs is that the MSCs need to be appropriately maintained in a viable and differentiated state along chondrogenic lineage in a microenvironment with chronic inflammation. Hydrogels are promising scaffolds with good biocompatibility and low immunogenicity to maintain the cell viability and prolong the retention of MSCs in the injection sites. As the stem cell’s fate is dictated by a complex interplay of biophysical and biochemical factors present in the native stem cell niche, one important direction of using stem cells for treating osteoarthritis in future will be the combination of direct modulation of stem cell behavior by gene delivery, gene editing with external factors such as growth factors or biomolecules provided by advanced hydrogels. This synergy approach will offer tremendous benefits over just relying on the scaffolds and signals alone and accelerate the application of MSCs in cartilage repair in osteoarthritis in future.

## Figures and Tables

**Table 1:** Comparison of MSCs behavior with different delivery method.

Delivery Method	Characteristic
Direct in vivo gene injection or indirect in vitro	Imprecise and transient transgenic expression, harmful side effects (immune response synovial cartilage formation)
Direct delivery using the combination of vectors and matrix	Efficient and durable cell transfection, regulation of pathological microenvironment
CRISPR/Cas9-mediated gene editing	Able to achieve ‘designer cells’ with feedback functions, e.g., regulation of inflammatory signal transduction, expression of anti-inflammatory drugs

## Data Availability

The datasets used and analyzed during the current study are available from the corresponding author on reasonable request.
